# Total sleep time, sleep efficiency, and next day subjective sleepiness in a large group of women

**DOI:** 10.1093/sleepadvances/zpac028

**Published:** 2022-09-07

**Authors:** Torbjörn Åkerstedt, Johanna Schwarz, Eva Lindberg, Jenny Theorell-Haglöw

**Affiliations:** Department of Clinical Neuroscience, Karolinska Institute, Stockholm, Sweden; Stress Research Institute, Department of Psychology, Stockholm University, Stockholm, Sweden; Stress Research Institute, Department of Psychology, Stockholm University, Stockholm, Sweden; Department of Clinical Neuroscience, Karolinska Institute, Stockholm, Sweden; Department of Medical Sciences, Respiratory, Allergy, and Sleep Research, Uppsala University, Uppsala, Sweden; Department of Medical Sciences, Respiratory, Allergy, and Sleep Research, Uppsala University, Uppsala, Sweden

**Keywords:** sleepiness, sleep efficiency, PSG, TST, sleep quality, anxiety, depression

## Abstract

The relationship between sleep duration and sleepiness has seen much research, but no data are available on the association between polysomnographically (PSG) determined total sleep time (TST) (or other PSG variables) and subjective sleepiness during the subsequent day in individuals in their habitual life situation. The purpose of the present study was to study the association between TST and sleep efficiency (SE) (and other PSG variables) and next-day sleepiness at 7 times of the day. A large population-based group of women (*N* = 400) participated. Daytime sleepiness was measured with the Karolinska Sleepiness Scale (KSS). The association was studied through analysis of variance (ANOVA), as well as regression analyses. For SE there was a significant difference in sleepiness across groups with >90%, 80%–89.99%, and <80% SE (F = 7.2, *p* < .001, eta2 = 0.04), with lowest sleepiness in the first group. In contrast, TST groups of <6 h, 6–6.99 h, and ≥7 h did not differ significantly. In addition, a pronounced U-shape (eta2 > 0.45) was seen for both analyses, with maximum sleepiness at bedtime (≈ 7.5 KSS units). A multiple regression analysis, including all PSG variables (adjusted for age and BMI), showed that SE was a significant predictor (β = 0.16, *p* < .05) of mean sleepiness, even after depression, anxiety, and subjective sleep duration were entered, but this was eliminated by subjective sleep quality. It was concluded that high SE is modestly associated with lower next-day sleepiness in women in a real-life context, but that TST is not.

**Statement of Significance**

High SE (in women) is associated with modestly lower sleepiness during the next day, compared to low SE, whereas differences in total sleep time or other PSG parameters are not. This should influence the interpretation of objective sleep measures in terms of daytime sleepiness.

## Introduction

Sleepiness has been defined as a drive towards sleep [1], and may be described in physiological, behavioral, and subjective terms [2]. It has been identified as a major cause of accidents [3]. The regulation of sleepiness and performance has been the subject of large amounts of research and seminal work has been carried out by Dr. Dinges’ group. In particular, their most relevant work for the present paper, has focused on the effects of one week of cumulative sleep restriction [4], 2 weeks of cumulative sleep restriction [5], the effects of recovery sleep [6], and the role of interindividual differences [7], using the Psychomotor Vigilance Test as the outcome, but also subjective sleepiness.

**Figure 1.**
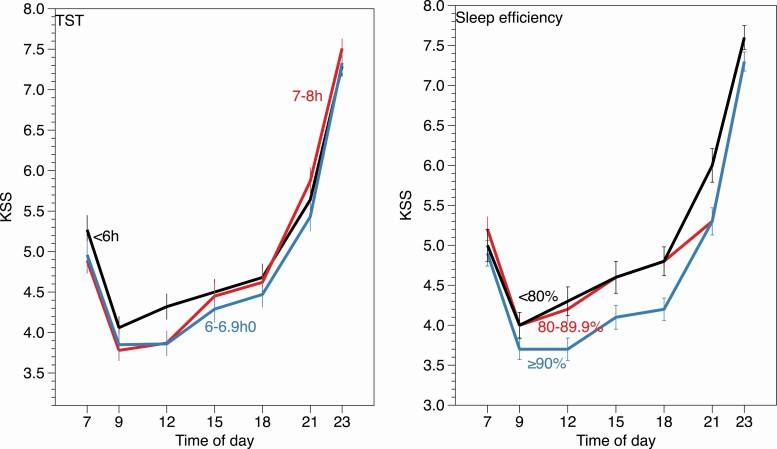
Sleepiness on Karolinska Sleepiness Scale (KSS) at different times of day for groups with different sleep efficiency (SE) (right) and total sleep time (TST) (left).

In relation to the present paper, it is of particular interest that the effect of the first restricted sleep with 5 h time in bed (TIB) in the cumulative sleep study showed a clear increase in subjective sleepiness (estimated from the figure) compared to 8 h TIB, as well as a modest increase across subsequent 6 days of restriction [4]. The results were similar with 4 h and 6 h across 2 weeks [8], and with 5 h, but not 7 h in a similar study [9]. Lo et al. showed a similar result, but with a nonsignificant reduction of subjective sleepiness after the first night with 6 h TIB (compared to 8 h TIB), together with strong cumulative effects from night 2 of sleep restriction [10]. Axelsson et al. found a clear (estimated from Figure 1) increase in subjective sleepiness after the first reduction to 4 h TIB [11], as well as a steady increase across the following days. Studies with one night of acutely restricted sleep did not show a significant increase in subjective sleepiness after restriction to 4 h [12, 13]. It is worth noting that 8 h TIB usually results in ≈ 7.5 h of total sleep time (TST), while 6 h usually yields ≈ 5.9 h. Overall, there seems to be a scarcity of larger dose-response studies on TST restriction and sleepiness.

The observations above raise the question of whether differences between individuals in objectively recorded sleep duration (TST) in a real-life context would show differences in subjective sleepiness during the subsequent day. Can one, for example, draw the conclusion that individuals with long objective sleep on a given day would show lower sleepiness the next day, compared to those with short sleep? This is a topic that has not been addressed before, and constitutes a different question from the experimental approaches. It also has a considerable applied value for interpreting sleepiness-related consequences of objectively recorded sleep. The research question requires that there is sufficient variation of TST across individuals to make it likely to find differences in sleepiness, and this seems to be te case [14, 15].

One may also wonder if it is objective sleep that has the closest link to next-day sleepiness, or whether subjective aspects of sleep duration, or perhaps sleep quality, may modify associations. In addition to TST, it might also be of interest to include sleep efficiency (SE), which is often rather highly associated with TST since SE represents TST/TIB.

This paper on sleep duration and sleepiness, uses the Karolinska Sleepiness Scale (KSS) as the outcome [16]. It has been used in most of the studies cited in the second paragraph above, and, is very sensitive to sleep loss, time on task, night driving, etc. It has also been shown to predict lane crossings in real (and simulated) driving [17], suggesting KSS >7 to constitute a criterion of risk.

The purpose of the present study was to use a large sample of women from the general population to study the association between different levels of TST, as well as SE, and the development of subjective sleepiness across the next day. Not only differences in levels were of interest, but also possible interactions; would, for example, low levels of TST or SE predict a steeper increase in sleepiness with increasing time awake? Furthermore, it was of interest to investigate if other PSG variables might be associated with sleepiness, and if the subjective perception of sleep duration or sleep quality for the same sleep, and also indices of depression or anxiety, would modify the associations between objective sleep and subsequent subjective sleepiness. For comparison, we also included the widely used Epworth Sleepiness Scale (ESS) as an outcome [18]. As it measures habitual sleepiness it might be of interest to investigate if this measure has an association with PSG variables from a single night or with the KSS. No previous work exists on these topics.

## Methods

### Design and participants

The present study is part of the Sleep and Health in women (SHE) at the University of Uppsala. The focus was on obstructive sleep apnea and several metabolic health parameters in women. A representative sample of 10 000 women in the region of Uppsala (Sweden) responded to a questionnaire on sleep/health (response rate 71.6%). A random sample (*N* = 400) was drawn from the cohort for PSG recording and blood samples (snorers were oversampled). These participants had their sleep recorded during weekdays and responded to another sleep and health questionnaire [19]. The present data are partly a follow-up of the original study after 10 years. It includes 273 individuals from the original cohort, and an additional 127 to replace those lost during the 10 years [20]. The sample used in the present study comprised 400 participants. The study was approved by the Ethical committee of the University of Uppsala (Dnr 2011/244).

Table 1 shows background data and for the recorded sleep. The mean age in the sample was 59.0 years (range: 35–81 years). Most (91%) were employed and married/cohabiting (69%). Among the PSG variables, 24 were lost due to artifacts affecting the computerized analysis. It is noteworthy that TST had a mean±standard deviation or 390 ± 73 min, and the corresponding values for SE were 85.2 ± 11.0%.

**Table 1.** Background data and overall results for recorded night

Background	*N*	Mean±SD or %
Age, y	399	59.0 ± 11.2
BMI, kg/m^2^	400	26.8 ± 4.8
Married/cohabiting	400	69%
Employed	398	91.0%
SRH, rather/very poor health	398	18.3%
Any alcohol ≥1/mo	396	29.0%
Smoker now (yes)	399	9.3%
Depression HAD (0–21)	400	4.51 ± 3.04
Anxiety HAD (1–28)	400	6.91 ± 3.72
KSS mean (1–9)	366	4.96 ± 1.07
ESS (0–21)	400	8.02 ± 4.33
Habitual sleep duration, h	367	7.10 ± 1.23
Habitual need for sleep, h	377	7.80 ± 0.87
Heavy snoring ≥ 3 d/week	385	22.1%
*Recorded night*		
TIB, min	373	460 ± 65
TST, min	374	390 ± 73
Sleep eff, %	371	85.2 ± 11.0
Awakenings/h	373	2.44 ± 1.50
Sleep latency, min	374	14.6 ± 24.5
N3 latency, min	370	35.8 ± 44.1
REM latency, min	367	119.4 ± 72.2
N1 %	374	17.4 ± 8.3
N2 %	373	50.1 ± 8.9
N3 %	374	15.6 ± 8.4
REM %	374	16.9 ± 7.5
AHI/h	367	19.8 ± 19.8
AHI/h ≥ 15	180	51.0%
AHI/h 0–14.999	187	49.0%
Subjective sleep duration, min	367	425 ± 73
Sleep quality (poor1−5good)	397	3.05 ± 1.08
Poor+ very poor sleep quality	397	37.3%
Sleep medication (yes)	397	5.3%

BMI: Body Mass Index; SRH: subjective health; HAD: Hospital Anxiety and Depression; KSS: Karolinska Sleepiness Scale; ESS: Epworth Sleepiness Scale; TIB: Time In Bed; N1-3: Stage N1, N2, and N3 sleep, respectively; REM: Rapid Eye Movement; AHI: Apnea-Hypopnea-Index

### PSG recording

Unsupervised sleep was recorded in the homes of the participants, who were asked to maintain their normal sleep hours. Embla solid-state, portable, sleep recording devices were used for recording sleep. Standard electrode (silver/silver chloride) montage included C3, C4 positions, referenced to contralateral mastoids. In addition, two submental electrodes, as well as electrodes at the outer canthi of the eyes were used. To adapt to AASM scoring, F4 was interpolated. In addition, data were obtained from, bilateral anterior tibialis muscles, airflow with a three-port oro-nasal thermistor and a nasal flow pressure sensor, respiratory effort from piezo-electric belts (Resp-EZ, EPM Systems Midlothian, VA, USA), finger pulse oximetry (Embla A10 flex Sensor), electrocardiograms (V5), a piezo vibration sensor for snoring and a body position sensor. These data are not reported on here in any detail. In the early evening, a researcher applied the electrodes, connected the equipment, and gave instructions. The equipment was retrieved the next morning by the researcher. Diary information was used to establish lights out and lights on.

Sleep scoring (stages, respiration, and arousals) were performed according to the classification criteria of the American Academy of Sleep Medicine [21], using the computer-assisted sleep classification system Somnolyzer 24 × 7 [22, 23]. Here the terminology N1, N2, and N3 is used for sleep stages 1–3. Wake after sleep onset (WASO) represents time awake between sleep onset and offset in minutes and is expressed as a percent of the total sleep period (TSP). Shifts from any of the sleep stages to wake are expressed as awakenings per hour. An apnea was defined as a cessation of airflow for at least 10 s, while a hypopnea was defined as at least 10 s of 50% reduced respiratory volume, together with at least 3% desaturation. The apnea-hypopnea index (AHI) was defined as the mean number of apneas and hypopneas per hour of sleep.

### Ratings

The main outcome was the score on the Karolinska Sleepiness Scale (KSS) at different times of day during the day following the sleep recording [16, 17], as well as the mean KSS for that day. The KSS ranges from 1 = extremely alert to 9 = extremely sleepy, fighting sleep, and difficult to remain awake. Also, the Epworth Sleepiness Scale (ESS) was used as a secondary outcome. It ranges from 0 to 24 [18].

Subjective sleep was rated using the Karolinska Sleep Diary after awakening and the items of interest were “sleep duration”, “sleep quality” (1 = Very poor to 5 = very good), “refreshed upon awakening” (1 = Not at all to 5 = Completely), and use of sleep medication for the recorded night (yes/no). Background items included were: age, rated subjective health rating (SHR, 1–5, very good–very poor), marital status (married/cohabiting vs living alone), employed (yes/no), BMI (weight/height^2^), any alcohol intake ≥1/month, smoking (Yes/No), habitual sleep duration, and heavy snoring ≥3 days/week vs. less. Also, the Hospital Anxiety and Depression scale (HAD) was included [24]. It contains seven items on depression and anxiety, respectively. Each item is scored 0–3, with a total of 0–21 for each scale.

### Statistics

For background data, mean ±standard deviation (SD), or %, were computed for all variables.

In order to study differences between amounts of TST and SE on the one hand and KSS at different times of day, on the other, an analysis of variance (ANOVA) with repeated measures was used, with high, medium, and low values on the predictors as a between-groups-variable. The main focus was on the difference between groups and possible interactions between groups and the time of day. The times of day were (within-participant): rising, 09:00 hours, 12:00 hours, 15:00 hours, 18:00 hours, 21:00 hours, and bedtime. For rising and bedtime, values were plotted at 07:00 hours and 23:00 hours. The analyses were adjusted for age.

In order to study if other PSG variables or ratings would be associated with mean KSS after the recorded sleep (or ESS) we first reduced the number of (frequently intercorrelated) PSG variables by computing correlations between all PSG variables and mean KSS across the day after the PSG recording. The significant variables were then entered into a stepwise multiple regression analysis with mean KSS as the dependent variable. The significant variables from that analysis were then entered, together with the significant ratings, adjusted for age and BMI, into a hierarchical multiple regression analysis, using as a dependent variable the mean KSS for the day after the recorded sleep.

## Results

### PSG groups and KSS at different times of day

For SE we selected three groups defined as having a SE ≥ 90% (*N* = 74, mean±se = 93.6 ± 2.3%), 80%–89.99% (*N* = 110, mean±se = 85.8 ± 2.7%), and <80% (*N* = 121, mean±se = 69.2 ± 9.7%), based on common notions in sleep research of high and low SE, although there is no formal consensus [25]. TST groups were divided into <6 h (*N* = 94, mean±se = 5.3 ± 0.6h), 6–6.99 h (*N* = 110, mean±se = 6.5 ± 0.3h), and 7–8 h (*N* = 101, mean±se = 7.7 ± 0.5 h), again based of common views and consensus on short and acceptable durations of sleep. No participant slept longer than 8 h [26].

Table 2 shows a significant variation of KSS across time of day for both variables and shows a pronounced U-shape, with highest sleepiness (≈ 7.5 KSS units) at bedtime (23:00 hours) (Figure 1), low values between 09:00 hours and 18:00 hours, and intermediate values at awakening and at 21:00 hours. Only SE showed a significant difference across groups. No interactions between the group and time of day were significant. Pairwise analyses showed significant (*p* < 0.05) lower sleepiness in the high-efficiency group, compared to the lowest efficiency group. We also investigated the possibility of an interaction between AHI and SE groups on mean KSS. The interaction was not significant, however (*F* = 1.24, ns), and neither was that for TST (*F* = 1.58, ns).

**Table 2.** Results from ANOVA, time of day and group for the three variables and their three levels. F-ratios, *p*-values

Variables	Time F-ratio	Group F-ratio	T*G F-ratio	Difference high vs. low group, *p*
SE	215.0***	7.2***	1.2	<.01
TST	245.8***	1.0	2.9	>.05
N1%	250.5***	1.6	1.0	>.05
REM%	238.1***	0.1	1.7	>.05
Df (range)	6/1812**–**1824	2/303**–**304	12/1812**–**1824	

*p < .05, **p < .01, *** *p* < .001. eta2 for time: 0.52 for sleep efficiency (SE), 0.45, for total sleep time (TST), 0.44 for REM%, and 0.46 for N1%. Eta2 for group: 0.05 for SE and <0.02 for the other three variables. Eta2 for interaction <0.02 for all variables. Adjustment for age.

Since KSS values of 8 or 9 are considered risk values (high sleepiness) [17], we also compared the three groups of TST and SE with respect to the presence of such values, using Chi2. No significant differences between groups were obtained for any of the time points, but the percentage of individuals of the total sample with high sleepiness was 7.2% for ratings at awakening, 1.1% at 09: 00 hours, 3.8% at 12:00 hours, 5.4% at 15:00 hours, 6.1% at 18:00 hours, 18.8% at 21:00 hours, and 54.5% at bedtime.

In order to shed more light on the subgroups of the analysis above, we compared the groups in the SE analysis, as well as the TST analysis, on several other variables possibly associated with sleepiness. These included AHI, age, coffee consumption, and sleep deficit (need for sleep–TST). This was done using analyses of variance (unadjusted) across groups.

For AHI and age, there was a significant difference across SE and TST groups, respectively (Table 3). AHI increased with decreasing SE and TST. Age decreased with increasing SE and decreasing TST. The significant association between groups and AHI disappeared when age was adjusted for (*F* = 1.95, ns for TST and 1.21, ns, for SE). The F-ratio for the number of cups of coffee was not significant *F* = 0.4, ns, (mean±se=2.1 ± 0.2 cups/day) for SE groups, and *F* = 1.7, ns, for TST groups (same mean±se). The sleep deficit for the whole sample (sleep need–TST) was 45.0 ± 60.3 min (mean±SD), but the groups did not differ significantly (*F* = 1.2 [ns] for TST, and *F* = 2.9 [ns] for SE).

**Table 3.** Results from ANOVA, SE and TST groups versus AHI and age. *F*-Ratios, *p*-values

SE groups	F-ratio and *p*	<80%	80%−89.99%	≥90%
AHI/h	3.80*	25.0 ± 2.2	20.0 ± 1.8	17.6 ± 1.6
Age, years	26.0***	63.5 ± 1.2	60.1 ± 1.0	53.6 ± 0.9
TST groups		<6 h	6–6.99 h	7−8 h
AHI/h	3.2*	23.7 ± 1.9	20.0 ± 1.7	17.0 ± 1.8
Age, years	15.8***	67.2 ± 1.1	58.6 ± 1.0	54.1 ± 1.0

*p < .05, **p < .01, ***p < .001. eta2 for time: 0.52 for sleep efficiency (SE), 0.45, for total sleep time (TST), 0.44 for REM%, and 0.46 for N1%. Eta2 for group: 0.05 for SE and <0.02 for the other three variables. Eta2 for interaction <0.02 for all variables. No adjustment.

### Prediction of mean sleepiness level during the day

Correlations were first computed between KSS and all PSG variables. This yielded significant correlations for SE (*r* = −0.14), TST (*r* = 0.13), N1% (*r* = 0.12), and REM% (*r* = 0.12), all significant at *p* < 0.05. Awakenings, sleep latency, N3 latency, REM latency, N2%, N3%, and AHI, were not significantly associated with mean KSS (*r* < 0.10, *p* > 0.05). To identify PSG variables for inclusion in the hierarchical multiple regression analysis we carried out a stepwise multiple regression analysis for the significant PSG variables with mean KSS as the dependent variable. Only SE became significant (β = −0.14, *p* < 0.05).

Correlations were computed between mean KSS and other variables, and yielded for age: *r* = −0.05 (ns), BMI: *r* = 0.05 (ns), depression: *r* = 0.24 (*p* < .001), anxiety: *r* = 0.20 (*p* <0.001), rated sleep duration for the recorded night: *r* = 0.22 (*p* < .001), rated sleep quality for the recorded night: *r* = −0.36 (*p* < .001), and sleep medication for the recorded night: *r* = 0.06 (ns).

The significant PSG variable(s) and ratings of the subjective duration of the recorded sleep, sleep quality of the recorded sleep, and intake of hypnotics before the recorded sleep were entered into a hierarchical multiple regression analysis. The analyses were adjusted for age and BMI. Table 4 shows that SE was a significant predictor of mean KSS, also after depression, anxiety, and subjective sleep duration were added. However, the significant contribution was lost when sleep quality was entered, and this variable became the strongest predictor, with subjective sleep duration and depression still remaining significant.

**Table 4.** Results from multiple regression analyses with age, PSG, and subjective sleep variables as predictors of mean KSS after the PSG recorded night. Beta coefficients for each model

Model no: *N*:	M1 321	M2 321	M3 299	M4 299	M5 299
Sleep eff.	−0.17^†^	−0.16^†^	−0.16^†^	−0.13*	−0.10
HAD anxiety		0.21^‡^	0.13*	0.08	0.06
HAD depression			0.12*	0.16*	0.15*
Subj sleep duration				−0.22^‡^	−0.12*
Sleep quality					−0.29^‡^

M = model = one predictor added for each model. Subj. = subjective. M = model.

*p < .05,

^†^ *p* < .01,

^‡^ *p* < .001. Adjusted for age and BMI.

We also analyzed the association between ESS and the same predictors as above, but none of the correlations with PSG variables were significant. Thus, no hierarchical regression analysis was carried out. The correlation between ESS and mean KSS was *r* = 0.20, *p* < .05.

TST for the recorded night was significantly associated with subjective sleep duration for the same night (β = 0.48, *B* = 0.48, *p* < .001), that is, for each minute of the increase in TST, the reported duration increased by 0.48 min (with a constant of 203 min).

## Discussion

The highest levels of SE (≥90%) showed the lowest levels of sleepiness across the day after the recorded sleep. A strong U-shaped pattern for sleepiness was seen across the day for SE, and TST. SE was the best PSG predictor of mean daytime sleepiness, even after depression, anxiety, and subjective sleep duration were entered into the hierarchical regression. However, entering subjective sleep quality eliminated the significant prediction.

The time-of-day effects in the present study are very similar to previous work on a large group of individuals during a regular working week [27]. It is also similar to results from laboratory studies [28]. The eta2 values indicate that the time-of-day effect on sleepiness was far greater than the relatively modest differences in SE between the groups.

The lower sleepiness across the day in the high SE group was hypothesized for logical reasons, but comparable data are lacking. The hierarchical regression analysis showed that SE maintained a significant association with mean sleepiness, even after variables like depression, anxiety, and subjective sleep duration were entered. This suggests a certain robustness. The significant association was lost only when sleep quality was entered, which may mean that the latter variable represents an overall perception of the total “goodness” of sleep. In addition, high SE maintained its difference from lower efficiency throughout the day. It is noteworthy that the regression analyses did not identify any other PSG parameters as predictors of next-day sleepiness.

One may wonder if the difference in sleepiness between SE groups may have practical implications for performance, and particularly, for safety. With respect to the latter, it has been shown that being taken off the road (by a driving instructor) for dangerous driving on the motorway in late at night occurred in 42% of the drivers, and at a mean sleepiness level of KSS = 8.5 (all ratings ≥8) during the preceding 5 min [29]. In real highway driving [30], or simulator driving [31–34], the probability of a lane departure is increased only at KSS levels 8 and 9, with very low probabilities at KSS levels <7. Thus, there exists a threshold effect, with substantial risk at levels >7 and very low risk at lower levels. This suggests that inefficient or short sleep, as measured by PSG in the present study, is unlikely to constitute a safety problem during daytime (09:00–18:00 hours) since sleepiness levels are modest (KSS ≈ 4–5). Bedtime levels showed high KSS (54.5% of the sample rated KSS ≥8), but this was not mainly due to low SE (or short sleep), but rather to circadian regulation and time awake, established factors behind subjective, behavioral, and physiological sleepiness [35].

Also, shorter sleep in the present study was expected to show higher sleepiness across the day, but did not. We have no clear explanation of the absence of such an effect, but it might be the case that TST “merely” reflects sleep duration effects, whereas SE also reflects time awake, as well as sleep interruptions, which could lead to additional sleepiness. An additional possibility is that the amount of sufficient sleep to obtain reasonable alertness varies between individuals, as suggested by the item “need for sleep” in Table 1, which indicates a wide span of need for sleep. Moreover, previous work seldom finds clear effects of modest sleep reduction. Thus, one night of reduced sleep (from 8 h to 4 h TIB) showed no significant difference in sleepiness, and chronic sleep restriction [13], with 4 h or 6 h TIB across 2 weeks, showed only modest (not tested) increases of sleepiness the first night [5]. Similar observation was seen for 5 h TIB, but not for 7 h (TIB) [9]. Possibly, the range of TST in the present population of women was too small (means of 5.3 vs. 7.7 h for lowest and highest groups, respectively) to result in increased sleepiness, particularly considering interindividual differences in need for sleep in a population-based sample under normal uncontrolled living conditions. This also implies that variation between individuals in sleep duration in a population should not be exaggerated in terms of severity with respect to sleepiness and daytime functioning. Furthermore, we don’t know if the associations of predictors and sleepiness in the present study were acute effects or not. They may also reflect habitual states, traits, but this is not possible to determine from the present data. It should be emphasized that the present results are based on women. Men may show different results.

We also investigated possible confounding variables like the AHI, age, coffee consumption, and sleep deficit, but no such confounding was observed (adjusted for age). In particular, AHI has been linked to sleepiness using the ESS, although relatively modestly (Ulander et al. 2022), and individuals with self-reported snoring were oversampled in the present study. This probably led to the rather high mean ± SD of AHI (19.8 ± 19.8), but the SD indicates a reasonably large variation for studies of association. Thus, we don’t see AHI as a likely confounder in the present sample.

The ESS scale was tried as an indicator of sleepiness, but the correlation with PSG variables was nonsignificant. The latter seems logical since the ESS describes habitual reactions to soporific situations and cannot be expected to represent sleepiness on a specific day [36].

One limitation of the present study might be that we only had a cross-sectional sample for analysis. A second PSG recording would have been interesting. Yet, such an approach would have reduced the sample size by half, due to limited resources, and a large, cross-sectional sample was preferred to a smaller one with repeated recordings. Another weakness may be the inclusion of women only. A male group would have added interest, and such work is underway. Still, women are the understudied sex in this type of research. Among the strengths of the present study, is that the sample was large and diverse and is representative of the women in the Uppsala region in Sweden.

In conclusion, we have demonstrated that there is a modest link between SE and subjective sleepiness in a cross-sectional sample living in their normal life situation. The link with TST was not significant. However, the perception of sleep quality seems more important in terms of next-day sleepiness than objective PSG parameters.

## Notes

This paper is part of the David F. Dinges Festschrift Collection. This collection is sponsored by Pulsar Informatics and the Department of Psychiatry in the Perelman School of Medicine at the University of Pennsylvania.
